# A Grounded Theory of Interdisciplinary Communication and Collaboration in the Outpatient Setting of the Hospital for Patients with Multiple Long-Term Conditions

**DOI:** 10.3390/jpm14050533

**Published:** 2024-05-16

**Authors:** Emma A. Gans, Ursula W. de Ruijter, Agnes van der Heide, Suzanne A. van der Meijden, Frederiek van den Bos, Barbara C. van Munster, Janke F. de Groot

**Affiliations:** 1 University Center of Geriatric Medicine, University Medical Center Groningen, 9713 GZ Groningen, The Netherlands; s.a.van.der.meijden@student.rug.nl (S.A.v.d.M.); b.c.van.munster@umcg.nl (B.C.v.M.); 2 Knowledge Institute of the Dutch Association of Medical Specialists, 3502 GH Utrecht, The Netherlands; j.degroot@kennisinstituut.nl; 3 Department of Public Health, Erasmus University Medical Center, 3000 CA Rotterdam, The Netherlands; u.deruijter@erasmusmc.nl (U.W.d.R.); a.vanderheide@erasmusmc.nl (A.v.d.H.); 4 Department of Internal Medicine, Northwest Clinics, 1817 MS Alkmaar, The Netherlands; 5 Department of Gerontology and Geriatrics, Leiden University Medical Center, 2333 ZA Leiden, The Netherlands; f.van_den_bos@lumc.nl

**Keywords:** multimorbidity, interdisciplinary communication, interdisciplinary collaboration, grounded theory, complexity science, tailored care

## Abstract

Interdisciplinary communication and collaboration are crucial in the care of people with multiple long-term conditions (MLTCs) yet are often experienced as insufficient. Through the lens of complexity science, this study aims to explain how healthcare professionals (HCPs) adapt to emerging situations in the care of patients with MLTC by examining interdisciplinary communication and collaboration in the outpatient hospital setting. We used the constant comparative method to analyze transcribed data from seven focus groups with twenty-one HCPs to generate a constructivist grounded theory of ‘interdisciplinary communication and collaboration in the outpatient setting of the hospital for patients with multiple long-term conditions’. Our theory elucidates the various pathways of communication and collaboration. Why, when, and how team members choose to collaborate influences if and to what degree tailored care is achieved. There is great variability and unpredictability to this process due to internalized rules, such as beliefs on the appropriateness to deviate from guidelines, and the presence of an interprofessional identity. We identified organizational structures that influence the dynamics of the care team such as the availability of time and financial compensation for collaboration. As we strive for tailored care for patients with MLTC, our theory provides promising avenues for future endeavors.

## 1. Introduction

An increasing number of people live with multiple long-term conditions (MLTCs), defined as two or more chronic conditions. Similar to most other European countries, it is estimated that 32% of the Dutch population lives with MLTCs [1,2]. Prevalence increases with age, reaching 87% of people aged 75 years and older [2]. Due to an aging population, the prevalence of multiple long-term conditions will continue to rise [1]. It is widely recognized that patients living with MLTCs require tailored and integrated care, which extends beyond their concurrent diseases [1,3,4,5,6,7,8,9]. Tailored care considers not only the biomedical domain but consists of an integrated biopsychosocial approach taking physical- and cognitive functioning, psychosocial context, as well as personal goals, priorities, and preferences into account [5,6,8,9]. However, because care systems and guidelines are mostly focused on distinct diseases, patients currently receive care from multiple healthcare professionals (HCPs) based on disease-specific guidelines [1,3,4]. This fragmentation of care often leads to polypharmacy and higher care utilization [4]. In addition, living with MLTCs is associated with lower life expectancy, poorer quality of life, and adverse psychosocial and functional outcomes [1,10].

Numerous studies and guidelines describe, in broad terms, what tailored and integrated care for patients with MLTCs should entail, highlighting the integral role of interdisciplinary communication and collaboration [7,8,9]. Interdisciplinary care has already become common practice for various single diseases (e.g., breast cancer) where it has resulted in improved quality of care and patient satisfaction [11,12,13,14,15,16]. However, the current literature and guidelines provide limited insight into the processes of interdisciplinary communication and collaboration for patients with MLTCs, nor do they offer guidance on how to optimize it to meet this prerequisite for delivering high-quality, interdisciplinary, patient-centered care.

To understand how interdisciplinary healthcare teams interact and collaborate, scholars have introduced complexity science, particularly complex adaptive systems (CASs), as a framework [17,18,19,20,21]. A CAS is *“a collection of individual agents with freedom to act in ways that are not always totally predictable, and whose actions are interconnected so that one agent’s actions changes the context for other agents”* [18]. As an open system, membership is subject to change and agents can be members of several systems at the same time [17,18,19,20,21]. Agents’ actions are based on internalized rules (instincts, beliefs, and constructs) that arise from past experiences. These rules are not necessarily shared or made explicit among members [17,18,19,20,21]. Furthermore, CASs are nonlinear: a small change can cause extensive and unpredictable consequences [17,18,19,20,21]. Despite the inherent unpredictability, it is often possible to make statements about overall patterns in the system [18]. Change in a CAS emerges through self-organization driven by local interactions between agents that ripple throughout the broader system, rather than being imposed or externally driven [17,18,19,20,21].

**Figure 1:**
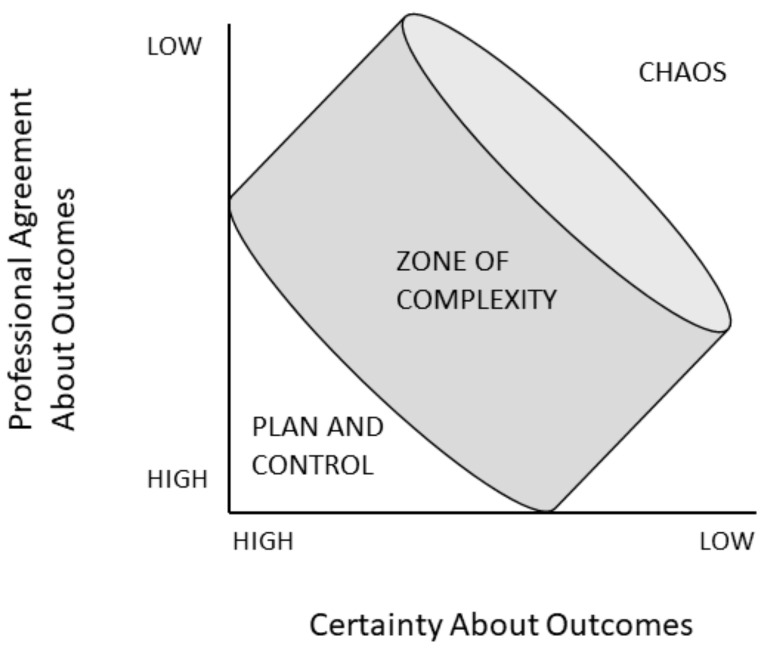
The certainty agreement diagram (based on [22]).

Adaptation in a CAS occurs when members interact with circumstances that fall in the “zone of complexity” (Figure 1) [22]. Circumstances with both a high level of agreement and certainty benefit from a hierarchical approach [18,19,21]. Circumstances with a very low level of agreement and certainty propel the system into chaos [18]. However, many situations in healthcare are in the complexity zone where there is a level of uncertainty about the best course of action, calling for a creative approach that requires continuous interaction between members, evaluation of the effect, and changing the course of action when appropriate [18,19,21]. In such cases, guidelines and protocols must be evaluated critically [19].

Research into the dynamics of healthcare teams using CAS principles has primarily focused on primary and nursing home care [23] and has not yet been applied to interdisciplinary care for patients with MLTC in the hospital. Through the lens of complexity science, the purpose of this study is to explain how HCPs adapt to emerging situations in the care for patients with MLTC by examining interdisciplinary communication and collaboration in the outpatient setting of the hospital. By doing so, we aim to understand how this can be optimized and offer guidance for future steps to improve coordination of care. 

## 2. Methods

### 2.1. Design and Data Collection

This descriptive qualitative study was based on constructivist grounded theory, a method used to explain the complexity of human interactions and social processes [24,25]. The theories are generated from the context in which they will later be applied [24]. In contrast to traditional grounded theory, constructivist grounded theory considers the researcher’s (theoretical) perspective as an integral role in the research process [26]. Through grounded theory, it is possible to move beyond the mere descriptive level of qualitative data by theorizing about actions and processes in a specific context, which aligns with the process-focused aim of our study. 

Data were collected using focus groups with physician specialists, physician assistants, and nurse practitioners involved in the care of patients with MLTC in the outpatient hospital setting. Focus groups help identify individual and shared ideas [27]. To ensure the expressed ideas were firmly rooted in daily clinical practice, we organized each focus group around a recent, local case study of a patient with MLTC and invited all HCPs of the care team of that patient to participate in the focus group. The focus group setting provided the opportunity for participants to interact and speak with candor about the process of their interprofessional communication and collaboration.

Participants were recruited through members of a national working group mandated by the National Society of Internists (NIV) to develop a guideline for coordinated care for patients with MLTC. Recruitment ran from October 2021 until June 2022. Working group members asked colleagues in their respective hospitals to provide case studies involving a patient with at least three chronic diseases and three HCPs in their outpatient care team. A minimum of three chronic diseases and three HCPs were chosen to ensure a sufficient level of complexity in communication and collaboration. For each case study, a treating HCP provided pseudonymized data on age, gender, number and type of chronic diseases and HCPs involved, number of hospitalizations, emergency room visits, and outpatient clinic visits in the previous year. Through purposive sampling, we selected patient case studies that involved a range of medical specialties from both secondary and tertiary referral centers. Through theoretical sampling after three focus groups, we also invited the primary care physician and/or nursing home elderly care physician. Focus groups were organized when at least three HCPs agreed to participate. When a participant could not attend the focus group after agreeing to participate, a separate interview was scheduled if possible. Demographic characteristics were collected for each participant (i.e., profession, gender, and age). Data collection ceased when no further insights emerged from the data [28].

Focus groups were held from November 2021 to July 2022 with EG, UdR, and JdG, and lasted 1.5 h. Focus groups were held online (Microsoft Teams or ZOOM) due to COVID-19 regulations. Attendees were instructed to find a comfortable, quiet room prior to logging on to prevent any distractions. All focus groups were led by an interviewer (EG) and supported by a note taker (UdR), both with a background in care for patients with MLTC. A senior researcher experienced in qualitative research and interdisciplinary organization of care (JdG) was present to observe, summarize, and ask follow-up questions. We started each focus group with an introduction of the case study by one of the participants. To help build rapport, we gave each attendee the opportunity to introduce themselves and how they were involved in the case study. Then we explored interdisciplinary communication and collaboration in relation to the case study using a discussion guide (Table 1). All focus groups were audio-taped for transcription.

### 2.2. Data Analysis

We analyzed the data throughout the data collection process according to the constant comparative method [24]. Following each focus group, all researchers present engaged in a brief discussion to establish findings that were similar across previous groups and findings that deserved consideration in upcoming sessions. All focus groups were transcribed verbatim by a team of research assistants. Transcriptions were checked by a researcher (EG) and subsequently approved by the participants of the respective focus group.

Data coding started after the first focus group was completed and was performed by two researchers (EG and UR). Open and axial coding were completed independently while making use of memos to conceptualize how codes relate. Discussion followed (EG and UR) to agree upon the core and sub-core categories. Both researchers analyzed the data once more using selective coding, focusing on the core and sub-core categories. This was not a linear process but involved constant comparison between data and the categories, actively looking for similarities and differences in the data. Next, two researchers (EG and UR) convened for multiple, extensive sessions to take the analysis to a higher level of abstraction by investigating potential theoretical relationships between categories and concepts in the data. This collaboration led to the development of the final theory, as described below in ‘results’. 

To enhance credibility and conformability, two independent researchers with experience in care for patients with MLTC performed the analysis. In case of no consensus, a third researcher with extensive experience in research on patients with MLTC (JdG) was consulted [29,30]. To ensure dependability, several experts working in the multimorbidity field performed skeptical peer review (JdG, AvH, and BvM) [29,30]. Finally, to strengthen credibility, we performed member checking by presenting the results to the mandated national working group including a representative of the Dutch Patient Federation [29,30]. All data were analyzed in Atlas.ti version 22. 

We report our findings in accordance with the Standards for Reporting Qualitative Research [31]. 

### 2.3. Ethical Approval

This study was approved by the Medical Research Ethics Committee of the University Medical Center Groningen (nWMO 202100785). Written informed consent was obtained from all participants. Pseudonymized data were analyzed, and data are presented in this paper anonymously.

## 3. Results

We conducted seven focus groups, with two to four participants in each session. One separate interview with a single HCP was held. Twenty-one HCPs participated, comprising fourteen physician specialists, two nurse practitioners, two residents, a psychologist, a dentist gnathologist, and a nursing home physician. The represented disciplines are presented in Table 2. The sample included twelve men and nine women, and physician specialists’ years of practice ranged from 1 to 29 years. Three focus groups were held in a secondary referral center, and four in a tertiary referral center. Case study characteristics are presented in Table 3.

### A Theory of Interdisciplinary Communication and Collaboration in the Outpatient Setting of the Hospital for Patients with Multiple Long-Term Conditions

**Figure 2:**
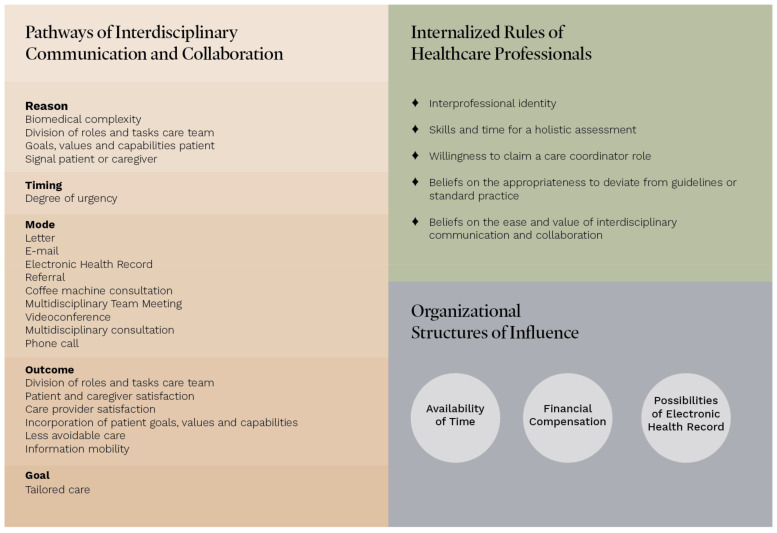
Main components of a theory of interdisciplinary communication and collaboration in the outpatient setting of the hospital for patients with multiple long-term conditions.

Our theory reveals the process of interdisciplinary communication and collaboration but does not predict how it will occur in a certain situation. The theory consists of three core components (Figure 2): ‘the pathways of interdisciplinary communication and collaboration’, ‘internalized rules of HCPs’, and ‘organizational structures of influence’. The ‘pathways of interdisciplinary communication and collaboration’ are separated into five parts: ‘reason’, ‘timing’, ‘mode’, ‘outcome’, and ‘goal’. Interaction between these parts is highly dynamic and influenced by ‘internalized rules of HCPs’ and ‘organizational structures’.

**Component 1: The pathways of Interdisciplinary communication and collaboration**

**I.**  **Reasons for interdisciplinary communication and collaboration**

The participants described situations in which they sought collaboration with their colleagues, which fall into four categories: ‘biomedical complexity’; ‘division of roles and tasks care team’; ‘goals, values and capabilities of the patient’; ‘signal patient or caregiver’. Commonly, participants sought collaboration for multiple reasons at once.

The first category, ‘biomedical complexity’, encompasses situations where HCPs are faced with a problem that falls outside of their own medical expertise, or where their own treatment or diagnostic decisions impact those of others. One participant explained that interdisciplinary communication allows them to *“call upon others to evaluate the situation too, because I can’t possibly know everything”.*

The second category, ‘the division of roles and tasks care team’, entails situations in which there is an unclear division of tasks and roles among HCPs. This often results in multiple HCPs attending to the same patient issue:

*“The primary care physician refers the patient to me [geriatrician], to assess the patient because of a multitude of problems. I run into complications of lung cancer and other comorbidities and start diagnostics and treatments. Simultaneously, the pulmonologist refers to the internists and the gastroenterologist to treat the same complications of lung cancer. Now, we have three people taking the lead on the same issues”*

Conversely, lack of communication may lead to issues not being attended to at all because HCPs assume that this falls under someone else’s responsibilities.

The third category, ‘goals, values, and capabilities of the patient’, refers to when HCPs aim to incorporate the individual patient’s goals, values, and capabilities into the treatment plan. This category also includes discussions on advance care planning and whether the treatment plan is proportionate to its goal, considering the context of the patient. Participants expressed that to do the above well and consistently across disciplines, interdisciplinary communication and collaboration are essential. However, as one participant described, HCPs often make assumptions rather than communicate:

*“The reason we sometimes, I think, make decisions with too little foundation, is that we see a high number of patients during short consultations. (…) Too often we assume that others, who refer the patient to us, have assessed whether the surgery is in line with the patient goals and values.”*

The fourth category, ‘signal patient or caregiver’, refers to instances where the patient or caregiver gives a direct or indirect signal that they experience fragmented care. A direct signal can be a patient who expresses that they received contradicting advice. An indirect signal may be fraught patient–doctor communication. One participant reflected on how she successfully organized a multidisciplinary team meeting for a patient who repeatedly reached out to her care team by e-mail and phone call, sometimes multiple times a day:

*“It [a multidisciplinary team meeting] gave them [patient and partner] a sense of peace, confidence that we are truly contemplating her problems, that we’re not abandoning them. We were able to confirm together as a care team that we don’t know all the answers yet. (…) She is now willing to wait and see how it goes.”*

**II.**  **Timing of interdisciplinary communication and collaboration**

Interdisciplinary communication and collaboration are most often employed for problem-solving, with varying degrees of urgency depending on the timing of interdisciplinary communication and collaboration. As HCPs wait longer to communicate or collaborate, problems become bigger or more acute. Participants expressed that collaboration early in the care process allows HCPs to prevent negative outcomes of care: *“Quite often, we consult the internist or geriatrician asking, ‘we have this and this in mind, is that possible?’”.* At the same time, participants expressed that the timing is often random and reactive:

*“It’s like ‘oh, something is wrong’, and now I must consult the internist, or the pulmonologist, or immunologist, or whatever. You notice it by chance because the patient is visiting your outpatient clinic at that time.”*

**III.**  **Mode of interdisciplinary communication and collaboration**

The participants described nine modes of communication and collaboration. There are four modes of indirect contact. First, ‘letters’ are written to update other HCPs, primarily those who work outside of the hospital. They simultaneously serve as comprehensive reports. Second, ‘e-mails’ are regularly exchanged when HCPs have a simple question. The advantages are their non-intrusive nature and minimal time investment. Disadvantages are that it is hard to keep track of unanswered e-mails and that not everyone replies promptly. Third, the ‘electronic health record’ is used to inform others by deliberately writing a more extensive report. Often-heard disadvantages were that these extensive reports can easily be overlooked, are difficult to find, and are limited to HCPs within the hospital. Fourth, HCPs make a ‘referral’ to colleagues when in need of their expertise or when they themselves experience too little time to take care of a problem outside of their usual tasks. Participants expressed that referrals are often utilized out of convenience because they require minimum effort. Participants conveyed that this behavior of *“stacking referral on top of referral”* is not desirable but happens frequently.

There are five modes of direct contact. First, participants expressed that they often consult a colleague haphazardly when they run into them, a mode we coined “coffee-machine consultation*”*. While this is experienced as convenient, the conclusions of these consultations are often not documented. Second, a ‘multidisciplinary team meeting’ is organized when it is deemed beneficial for more providers to speak to each other at the same time. A multidisciplinary team meeting for a patient with MLTC, with all involved HCPs, is not embedded in standard clinical practice. It can be organized ad hoc but this occurs rarely, because HCPs consider this time-consuming and logistically challenging. Third, a ‘video conference’ is sometimes organized instead of a multidisciplinary team meeting. This is considered especially convenient when HCPs from other organizations are invited to join. Fourth, ‘multidisciplinary consultations’ are standardized consultations where two professionals see patients together. For example, participants described oncologists and geriatricians holding clinic hours together. Fifth, ‘phone calls’ are made for relatively simple questions. Listed advantages are the minimal time investment and the possibility of an immediate answer. A disadvantage is that it is difficult to gauge when it is a convenient time to call.

**IV.**  **Outcome of interdisciplinary communication and collaboration**

Depending on the reason for -and chosen mode of- interdisciplinary communication and collaboration, participants described six outcomes that can be positively affected. However, when a reason for interdisciplinary communication and collaboration is present, but HCPs fail to act or choose an inappropriate mode, this can result in a lack of a positive impact or even a negative impact on these outcomes.

First, interdisciplinary communication and collaboration can provide clarity by making the ‘division of roles and tasks of the care team’ explicit. Participants described the importance of making deliberate choices concerning who should deliver which care and where it should take place:

*“It is essential to involve the primary care physician in the discussion concerning advance care planning, but also to discuss which tasks the primary care physician is responsible for.”*

Participants described finding it helpful to appoint someone within the care team as the coordinator of care. This coordinator is responsible for aligning care plans, communicating across all disciplines, and can serve as the point of contact for the patient.

Second, proper interdisciplinary communication and collaboration can improve ‘patient and caregiver satisfaction*’* and ‘HCP satisfaction’. Participants expressed that patients and caregivers value it greatly when their HCPs are aware of each other’s treatment trajectories. An often-heard complaint is that HCPs give contradictory advice. Communicating as a team can be beneficial:

*“It can be good for patients to hear the same story from different people. (…) A patient sometimes needs that, especially when there are many factors at play and a lot of pain.”*

Participants explained that proper interdisciplinary communication and collaboration can also lead to HCP satisfaction because the lack thereof is often experienced as frustrating.

Third, participants discussed that interdisciplinary communication and collaboration can aid in ‘incorporating patient goals, values, and capabilities’. Reflecting on the case studies, participants often expressed that, in hindsight, they would have liked to be aware of information concerning patient goals, values and capabilities that other health professionals had but they did not ask for:

*“She [the patient] has a history of posttraumatic stress disorder and a cognitive disability. What does that mean for her? What do we need to consider? In hindsight these are things I would have liked to know.”*

In this situation, neglecting to involve the answers to these questions in the patients’ care plan led to multiple, highly acute readmissions and unsafe circumstances at home without the needed support.

In addition, participants described ‘less avoidable care’ as a positive outcome. Participants were adamant that proper interdisciplinary communication and collaboration would lead to less avoidable and unnecessary care. Examples given were stopping medication or treatments earlier, avoiding protocolized care that does more harm than good and reducing the number of referrals. Participants also described how aligning care plans can prevent negative effects of drug and treatment interactions, such as emergency room visits or hospitalizations. Reflecting on the case studies, all participants described the provision of care that was considered unnecessary and avoidable due to a lack of interdisciplinary collaboration. For example, one surgeon described planning a surgery for a patient without being aware of comorbid dementia, for which the patient was receiving care in the same hospital:

*“The most important thing is that we didn’t operate the aneurysm and avoided looking back saying ‘mm, should we have done that…’. In that regard, it’s not too bad, but in terms of burden, hospital visits, diagnostics… those things we could have avoided for him if we approached it in an interdisciplinary way sooner.”*

Finally, closely intertwined with the outcomes ‘incorporation of goals, values, and capabilities’ and ‘less avoidable care’, is the outcome ‘information mobility’. Information mobility can help facilitate the former two outcomes. It refers to being able to access and share patient-related data and information, as well as other HCPs’ considerations and plans, seamlessly and securely within and across organizations. Participants described that as they work in silos, information tends to remain stagnant. Enhancing interdisciplinary communication and collaboration can lead to the improvement of information mobility, and vice versa.

**V.**  **Goal of interdisciplinary communication and collaboration**

The overarching goal for interdisciplinary communication and collaboration was described as tailored care. One participant described that, regardless of the number of HCPs involved, the provision of tailored care should be guaranteed, and a suitable manner to accomplish this should be found:

*“You should always try to tailor care to comorbidities, psychosocial context, and the goals, values, and priorities of the patient. It doesn’t matter if you’re the only treating physician or if there are many. And then it depends if it is necessary for this specific patient to gather everyone around a table for a discussion or if a more informal way of collaboration suffices. Like oh, I’m seeing this patient, my plan is this and that, does that align with your plan and goals?”*

**Component 2: Internalized Rules of HCPs**

Participants described five internalized rules that influence whether they follow up on a reason that calls for interdisciplinary communication and collaboration, which mode they employ, and, consequently, which outcomes they are likely to bring about.

First, participants expressed varying degrees of ‘interprofessional identity’*:* some find it more important than others to collaborate and engage in collaboration more often. Some HCPs described that they are attached to delivering disease-specific care, that they will not entrust to others:

*“Care coordination by a pulmonologist-oncologist is fine, but they should not meddle with heart failure medication. (…) Giving up control like that is not something easily done within my specialty.”*

Second, participants described that to reach outcomes such as “incorporating patient goals, values, and capabilities”, and “less avoidable care”, it is required to assess the patient in a holistic manner. Whether participants believed they had the ‘appropriate skills and time required to perform a holistic assessment*’* varied. Some believe these skills are exclusive to a small number of specialists:

*“Of course, there are a few general specializations, such as internists and geriatricians, but that’s about it. All other specialists work solely from the perspective of their specialization and sub-specialization.”*

Third, participants described that HCPs have varying levels of ‘willingness to claim a care coordinator role’:

*“What makes the system very fragile, is that it is very dependent on who the patient visits first in the hospital. (….) I think that many specialists, they don’t feel inclined to claim a care coordinator role, they don’t feel equipped, or they don’t have time.”*

The likelihood of experiencing a sense of duty to claim a care coordinator role increases when HCPs have treated the patient for a long time and know them well.

Another important internalized rule that influences the willingness to claim this role, is to what degree HCPs consider it ‘appropriate to deviate from nationally endorsed guidelines or standard practice’ when they believe it justified, and whether they are comfortable doing so. For example, some HCPs feel forced into action when colleagues refer to them or give advice:

*“Once a referral has been made, then, apparently, a general practitioner or a nursing home doctor is also in a difficult situation because of a family who believes ‘grandma should live until 100’ or whatever it may be. And then there’s unrest, and they come to us, and then you have to proceed.”*

Participants expressed that the years of practice experience influence whether HCPs are comfortable deviating from the usual course of action.

In addition, participants described their ‘beliefs concerning the ease and value of interdisciplinary communication and collaboration’*,* and the interaction between them. A factor that influences whether coordinating care with colleagues is experienced as easy is the degree to which an HCP knows their colleagues well and whether they are in proximity to each other:

*“I have two or three neurologists and a few cardiologists specialized in heart rhythm disorders that I can call. And well, among the internist, there are a few that I can approach.”*

How a mode of communication is perceived by HCPs, influences the likelihood of them using it. For example, if an HCP considers it too burdensome, or not their responsibility, to coordinate care with multiple HCPs by means of a multidisciplinary team meeting, they will refrain from coordinating care in this manner. However, when they see great value for a specific patient, or themselves, they will do so even if the time investment is significant.

**Component 3: Organizational structures of influence**

The way HCPs respond to emerging problems and choose to collaborate is influenced by several factors that relate to the broader healthcare system. Three factors were reiterated in all focus groups. First is the ‘financial compensation’, or lack thereof, for the different modes of communication and collaboration. For example, an HCP receives financial compensation for evaluating a patient that is formally referred to them, while engaging in an ad hoc multidisciplinary team meeting about the appropriate course of action is not compensated. A second factor is the extent to which HCPs have ‘flexibility in managing their daily schedules and time allocation’. For many, time is experienced as a scarce resource in their daily schedule and patient consultations are often limited to ten minutes. This limits HCPs greatly to what degree they can go beyond the minimal required effort in patient care. Finally, the ‘electronic health record’ is perceived as a barrier to interdisciplinary communication and collaboration because of the poor integration of patient information from other systems and the (un)findability of data. At the same time, HCPs see great potential in electronic health systems supporting them in collaborating across disciplines and hope that technological innovation will realize this.

## 4. Discussion

Our study illustrates that HCPs in the outpatient setting of the hospital behave as a CAS in the care for patients with MLTC. To achieve tailored care, they often need to adapt to emerging situations through interdisciplinary communication and collaboration. We offer a theory of this adaptation process that covers the reasons that can catalyze an adaptive response (i.e., problems within the zone of complexity), its different approaches and outcomes. The adaptive response is shown to be non-linear and unpredictable, and its outcome variable. Moreover, there are numerous internalized rules at play that influence HCP behavior. Finally, we identify limitations to the self-organizing abilities of the system due to the environment in which it operates. Our study adds to the current body of literature that explores interdisciplinary healthcare teams as a CAS and is the first to apply this to care teams of patients with MLTC in the hospital.

Compared to other interdisciplinary teams that have been studied as CAS in the literature, there are a few elements that are distinct to the interdisciplinary team for patients with MLTC. Until now, CAS research primarily focused on interdisciplinary teams in nursing homes or primary care [23], which exhibit high levels of stability as they work on common ground towards objectives that are often shared. Contrarily, for patients with MLTC, HCPs join and leave care teams continuously depending on patients’ comorbidities and developments in health status. HCPs can therefore be members of a multitude of differently composed interdisciplinary teams. Moreover, HCPs often work towards their own condition-specific goals, which are not shared across all members. Membership fluidity can be problematic to collaboration because it is known to reduce the feeling of belonging to a team, and can diminish trust in other team members to effectively complete the task [32].

Another element distinct to care teams for patients with MLTC is that there is no set moment for collaboration. Thus, this is heavily dependent on opportunities created by members of the care team and the way in which they do so. Our theory describes nine modes of communication that members employ to create such opportunities. Due to the non-linear nature of the system, it is impossible to assert that mode X leads to outcome Y. However, it needs to be underlined that not all forms of communication lead to true collaboration when they are used as an exclusive approach. For example, merely referring patients from one HCP to the next, does not sustain collaborative teamwork and is unlikely to contribute towards tailored care. Research by Dukewits and Gowan, described the presence of a collaborative culture, fostered by activities such as conducting effective team meetings and reflecting on team performance, to be essential to successful collaborative teams [33]. As shown in other clinical settings, just because people are members of the same team, does not mean that they behave as such [33,34]. Our study shows that the fluidity of the care team, in combination with the lack of a set moment for collaboration (i.e., regular team meetings) is likely to diminish team identity and collaborative behavior.

We identified six internalized rules that influence if, and to what degree, an HCP engages in interdisciplinary collaboration. Some of these internalized rules seem more pivotal to obtaining the goal of tailored care, such as ‘interprofessional identity’. The literature on interprofessional identity describes how a diminished team identity and team performance, as seen in highly fluid interdisciplinary teams, can be overcome when individuals identify themselves with a larger group of various professions [35,36]. A strong interprofessional identity has a positive effect on interprofessional collaboration [36]. A survey among HCPs in the Netherlands showed that, overall, the interprofessional identity of the different specialties is strong but the individual differences within groups are significant [37]. These individual differences contribute to the variability of the degree of collaboration that we describe in our theory.

‘Beliefs on the appropriateness to deviate from guidelines or standard practice’ is another key internalized rule in realizing tailored care. The literature shows that following disease-specific guidelines may be inappropriate, as these guidelines frequently do not apply to patients with MLTC, may be mutually incompatible, and may result in an increased treatment burden [1,3,4]. However, we found that not all HCPs are comfortable to deviate from guidelines or standard practice. This is in line with a study by Brown et al., which identified medicolegal vulnerability as a barrier for primary care physicians to deliver personalized care to older patients with MLTC [38]. This calls for a discussion and improved comprehension of the intricate challenges in providing care for patients with MLTC within the medicolegal domain.

Our theory also uncovers starting points to improve interdisciplinary communication and collaboration for patients with MLTC. The outcomes and goals we present in our theory show that HCPs believe that interdisciplinary communication and collaboration can lead to improved care for patients with MLTC, as described in guidelines and consensus documents [5,7,8,9]. At the same time, a key conclusion from our study is that whether the CAS adapts to emerging situations in such a way that the outcomes and goals are achieved, is highly unpredictable and dependent on members of the CAS, and their interrelationships and connections. Given that care teams for patients with MLTC behave as a CAS, it is important to accept that adaptive responses will never be linear and predictable. However, provided that tailored care is greatly beneficial to the patient, we should strive to consistently achieve this for patients with MLTC and thus reduce the variability in patient healthcare experiences.

Solutions in healthcare tend to be top-down and linear: implementing care pathways, defining roles and responsibilities, standardizing consultations, etc. Although these solutions have their own merits, we should also explore innovative solutions that foster collaborative care because interdisciplinary teams in the care for patients with MLTC behave as a CAS. When we recognize that the connectivity between members of a CAS is more important than the members themselves, it becomes clear that future efforts should focus on lifting barriers that halt this connectivity and on promoting behaviors that facilitate it. The internalized rules and structures of influence identified in our theory, provide anchor points for innovative endeavors. For example, medical curricula should cultivate a stronger interprofessional identity and focus on enabling HCPs to recognize which problems lie in the zone of complexity that ask for a different response than merely following guidelines and protocols. Secondly, providing time and financial compensation for efforts that promote tailored care will create the freedom for a CAS to self-organize and adapt to problems in an effective manner. Third, efforts to enhance information mobility and technological innovations for electronic health records could play an important role in facilitating tailored care. Another direction of interest is to take a closer look at patterns that emerge in a CAS. While CASs are intrinsically unpredictable, it is possible to draw conclusions from overall patterns that emerge [18]. For example, in the case studies discussed, one patient had 64 phone calls or visits to the outpatient clinic in the past year. While it is impossible to predict the timing or reason of the next consultation, it is evident that unless the system adapts, this patient will appear very regularly. Activating HCPs to look for these patterns could trigger adaptive responses where necessary. Providing evidence through future research endeavors to support that strengthening interdisciplinary communication and collaboration will reduce care utilization, alleviate treatment burden, and improve patient care experiences, would help in creating consensus amongst HCPs regarding the approach to care and expected outcomes.

An important limitation of our study is that although we aimed to apply purposive and theoretical sampling, we were limited to participants that volunteered to partake. The primary care physicians of the patients discussed in the focus groups, for example, were invited after three focus groups but were unable to join. Therefore, our performed sampling methods closely resembled convenience sampling, potentially leading to selection bias. It did however provide valuable insight because the difficulties we experienced in organizing a focus group study with all HCPs involved in the care for patients with MLTC are likely to resemble the difficulty of organizing an ad hoc multidisciplinary team meeting. We experienced the enthusiasm of most HCPs to engage in the group discussion concerning this topic, but also the restraints of time and lack of flexibility in their schedule.

Furthermore, our findings should be interpreted in the context from which our grounded theory emerged: secondary and tertiary hospitals in The Netherlands. The unique characteristics of our healthcare system, working environment, and culture may limit the broader generalizability to other healthcare systems or cultural contexts. At the same time, most Western healthcare systems face similar challenges concerning the fragmentation of care, so valuable insights can still be drawn from our findings while taking context into consideration.

In conclusion, interdisciplinary care teams in the outpatient setting of the hospital operate as a CAS in the care for patients with MLTC. Our theory elucidates the different pathways of communication and collaboration that exist in the care of patients with MLTC. Why, when, and how team members choose to communicate and collaborate influences if and to what degree tailored care is achieved. Currently, there is great variability and unpredictability in this process due to internalized rules and organizational structures. As we strive for tailored care for patients with MLTC, our theory provides promising avenues for future endeavors to optimize interdisciplinary communication and collaboration that take the principles of a CAS, such as non-linearity and self-organization, into account.

## Figures and Tables

**Table 1:** Discussion guide.

In what manner did interdisciplinary communication and collaboration play a part in the care for this patient? How would you define your role in the interdisciplinary collaboration for this patient? Were there situations exemplary for how interdisciplinary communication and collaboration should take place? Are there situations that could have benefited from stronger interdisciplinary communication and collaboration? Reflecting on situation X [specific situation presented in discussion of the case study], what led to interdisciplinary collaboration taking place/not taking place? Can you describe what the potential benefit would be for patients and health care providers in general if interdisciplinary collaboration and communication was strengthened for this group of patients?

**Table 2:** Disciplines represented in focus groups.

Discipline	Number of Participants in Focus Groups
Internal medicine	7
Geriatrics	2
Cardiology	2
Surgery	2
Orthopedic surgery	1
Rehabilitation medicine	1
Psychiatry	1
Anesthesiology	1
Neurology	1
Psychology	1
Dentistry	1
Nursing home elderly care	1

**Table 3:** Case study characteristics.

Gender (male (n); female (n))	3 male; 4 female
Age in years (mean, range)	69 (55–80)
Number of chronic illnesses (mean, range)	4 (3–7)
Number of health care professionals involved in the outpatient care team (mean, range)	5 (3–9)
Emergency department visits in the past year (median, IQR)	1 (0–3)
Hospitalizations in the past year (median, IQR)	2 (0–3)
Outpatient visits or phone calls in the past year (median, IQR)	38 (6–57)

## Data Availability

The participants of this study did not give written consent for their data to be shared publicly. So, due to the sensitive nature of the research, full transcripts are not available. Relevant excerpts are shared in the manuscript.
